# Tofacitinib for the treatment of tumor necrosis factor-α inhibitor refractory esophageal Crohn’s disease: a case report

**DOI:** 10.1186/s13256-016-1036-y

**Published:** 2016-09-23

**Authors:** Sunina Nathoo, William A. Hood, Sara Keihanian, Amy L. Collinsworth, Sarah C. Glover

**Affiliations:** 1 University of Florida, College of Medicine, 1600 SW Archer Rd, Room 4102, Gainesville, FL 32611 USA; 2 Department of Graduate Medical Education, Nova Southeastern University, Largo Medical Center, 201 14th St SW, Largo, FL 33770 USA; 3 Division of Gastroenterology, Hepatology and Nutrition, University of Florida, 2000 SW Archer Rd, Gainesville, FL 32610 USA; 4 Department of Pathology, Immunology, and Laboratory Medicine, University of Florida, 2000 SW Archer Rd, Gainesville, FL 32610 USA

**Keywords:** Esophageal Crohn’s disease, Dysphagia, Tofacitinib, Inflammatory bowel disease, Therapeutics

## Abstract

**Background:**

Esophageal Crohn’s disease is reported as a rare manifestation, although its prevalence may be underestimated because upper endoscopies are not routinely performed in asymptomatic adults. Tofacitinib, an oral janus kinase inhibitor, is a new biologic that has shown promise in the treatment of ulcerative colitis and may be effective in the treatment of Crohn’s disease according to phase 2 trials. We report the first case of esophageal Crohn’s disease successfully treated with tofacitinib in a patient with worsening symptoms despite maintenance therapy with a tumor necrosis factor-α inhibitor.

**Case presentation:**

A 67-year-old Caucasian woman presented with new dysphagia and had findings of esophageal Crohn’s disease on endoscopy. The dosage of her current biologic therapy—adalimumab—was increased in frequency, without improvement. Our patient was started on tofacitinib and demonstrated an improvement in symptoms, with a repeat endoscopy showing resolution of the previous lesions.

**Conclusion:**

Esophageal Crohn’s disease is likely underdiagnosed but is an important consideration in a patient with new symptoms of dysphagia and known Crohn’s disease. Tofacitinib, while a novel agent, could have a role in the treatment of esophageal Crohn’s disease that does not improve with intensification of the current biologic therapy. It provides a different mechanism in patients who become refractory to maintenance therapy.

## Background

Esophageal manifestations of Crohn’s disease (CD) include erosion, ulceration, stricture, and fistula formation. Patients can present with odynophagia and/or dysphagia, but more serious manifestations including bronchoesophageal fistula have been reported [1]. Involvement of the esophagus in CD is rare and it is the least commonly affected segment in CD [1]. Additionally, the lack of standard upper endoscopy in asymptomatic adult patients with CD could also contribute to its low incidence [2]. Therefore, the prevalence of esophageal Crohn’s disease could be underestimated; it is currently reported as being 1–6.8 % in the literature [1–4]. In contrast, there is a higher incidence of esophageal CD reported in the pediatric population because screening upper endoscopy is performed at the time of diagnosis given the high degree of proximal active disease, although patients are typically asymptomatic [2]. However, new studies suggest that upper gastrointestinal involvement is seen equally in the adult and pediatric populations [5]. There is a lack of consensus in the definition of involvement and, until recently, on treatment [4, 5]. However, recent guidelines suggest treatment with immunomodulators, and tumor necrosis factor-α (TNF-α) inhibitors if the disease is more refractory; both options have been described in several case series with success [2, 6, 7]. We report the first case of esophageal CD successfully treated with tofacitinib, an oral janus kinase (JAK) inhibitor, in a patient with known CD. To the best of our knowledge, this is the first reported case of its use in esophageal CD. We also review the presentation, diagnosis, and treatment of esophageal CD.

## Case presentation

A 67-year-old Caucasian woman with a history of small and large bowel CD presented with new dysphagia. She was diagnosed with CD at age 30. Her disease had been complicated by fistula formation and multiple small bowel resections, as well as small intestinal bacterial overgrowth, chronic diarrhea, and osteoporosis. At the time of presentation, she had been on adalimumab (40 mg every other week) and mesalamine for 2 years. Previous medications included 6-mercaptopurine, azathioprine, methotrexate, infliximab, prednisone, and budesonide. She was not on a proton pump inhibitor at the time. Her vital signs were all within normal limits and her physical examination was unremarkable.

**Fig. 1:**
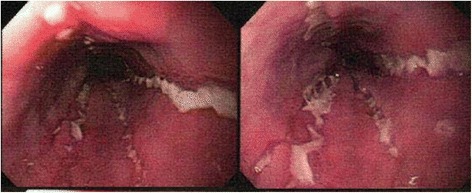
Endoscopic findings of longitudinal, serpiginous ulcerations along the entirety of the esophagus consistent with an esophageal manifestation of Crohn’s disease. Our patient was on adalimumab and mesalamine at the time

**Fig. 2:**
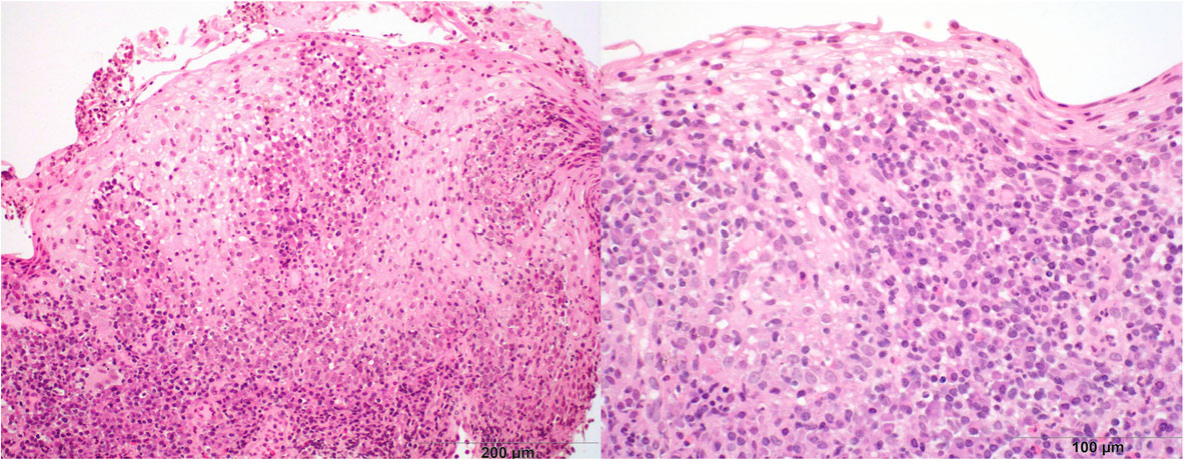
Esophageal biopsies from an initial endoscopy that show granulomatous changes on low (*left*) and high (*right*) magnification

An upper endoscopy was performed and revealed deep linear ulcerations throughout the majority of her esophagus (Fig. 1) with a normal stomach and duodenum. Esophageal biopsies showed lymphocytic esophagitis characterized by dense lymphoplasmacytic infiltrates within the mucosa and lamina propria and an ill-formed granulomatous reaction consistent with an esophageal manifestation of CD (Fig. 2). Results from staining for acid-fast organisms with acid-fast bacillus (AFB) stain were negative and no viral cytopathic effects or intra-cellular inclusions were found. Our patient also had negative results in an interferon-gamma (IFN-γ) release assay, no remarkable findings on a chest X-ray, and computed tomography enterography that was negative for any intraluminal masses.

Our patient was subsequently started on a budesonide suspension but had an inadequate clinical response after 5 months. Thus, adalimumab was increased to 40 mg every week. A repeat endoscopy showed mid-esophageal ulcerations that were biopsied. Examination of the specimens demonstrated inflamed granulation tissue and inflammatory exudate. Immunohistochemical stains of the biopsy specimens were negative for herpes simplex virus and cytomegalovirus. At this point, omeprazole 40 mg once daily was started, but her symptoms persisted despite intensified TNF-α inhibitor therapy and concurrent proton pump inhibitor use. Adalimumab was thus stopped. Tofacitinib 5 mg twice daily was started after consideration of both ustekinumab and a drug trial involving an interleukin (IL) 6 inhibitor. Ustekinumab use was precluded by insurance and cost. Vedolizumab had not yet been approved for clinical use.

**Fig. 3:**
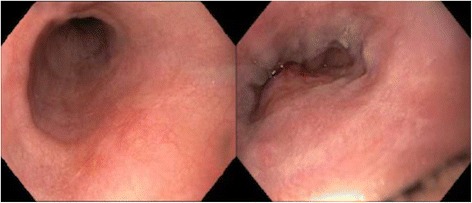
Repeat endoscopy shows healed esophageal mucosa following initiation of tofacitinib

**Fig. 4:**
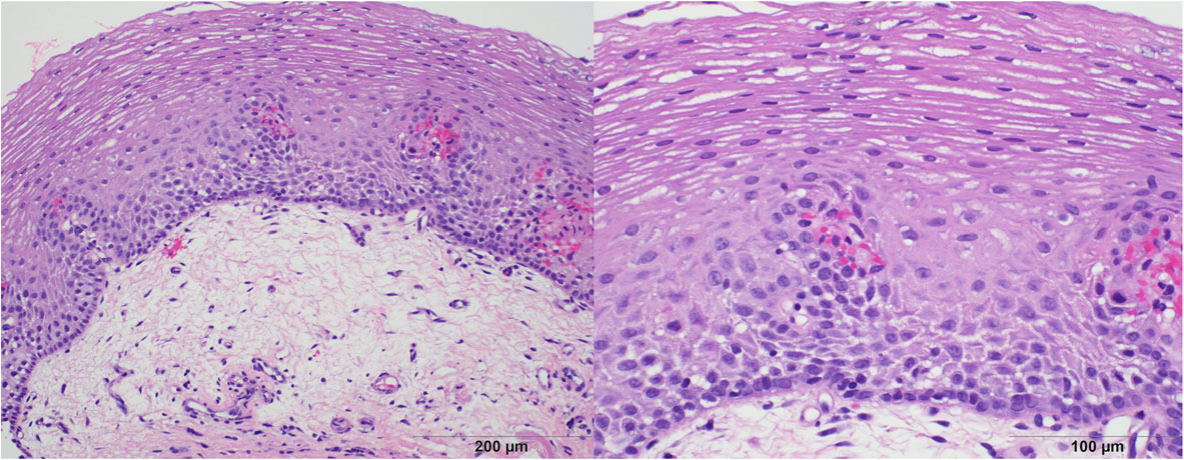
Esophageal biopsies after treatment that show a normal esophagus without significant alteration on low (*left*) and high (*right*) magnification

Six months later our patient was seen in a follow-up appointment and had no esophageal symptoms. A repeat endoscopy with biopsies was performed 7 months after she started taking tofacitinib. This study demonstrated esophageal scarring and nonspecific chronic inflammation in her distal esophagus, but showed complete resolution of her previous ulcerative disease (Fig. 3). Biopsies demonstrated that her mucosa showed no significant granulomatous changes as were previously found (Fig. 4). A repeat endoscopy 2 years after tofacitinib initiation was unremarkable and she did not experience any adverse events.

## Discussion

There are several presentations of esophageal CD that the reader should be made aware of. These include dysphagia or odynophagia as seen in our patient due to deep ulcers, strictures with or without fistula in an older patient, or asymptomatic in patients with normal endoscopy results but abnormal histology [1–3]. The latter is seen in pediatric patients, who have a high prevalence of esophageal CD when surveyed with upper endoscopy [1]. Patients may also present with chest pain, heartburn, nausea, and vomiting [8]. Almost all cases are seen with CD affecting other segments of the gastrointestinal tract, although isolated esophageal involvement has also been reported [3, 9]. Diagnosis is aided by endoscopy but requires an integration of clinical, endoscopic, histological, and radiographic data [2, 3]. Findings on endoscopy include aphthous ulcers, superficial erosions, friability, and nodular thickening [3]. The importance of biopsies cannot be underscored enough given the broad differential diagnosis, although histological findings may not be specific for CD, making the diagnosis difficult [2]. The transmural nature of CD is also a factor, which cannot be assessed with superficial biopsies taken during endoscopy. Histological findings include chronic, non-specific inflammation with or without granulomas, although these are rarely observed [5, 8, 10]. Granulomas are seen in only 20–30 % of grossly abnormal biopsies but are considered pathognomonic for CD diagnosis [4].

While our patient had histological evidence of esophageal CD, the differential diagnosis would also include reflux esophagitis, pill esophagitis, viral esophagitis (cytomegalovirus and herpes simplex virus), monilial esophagitis, granulomatous diseases such as sarcoidosis, tuberculosis, necrotizing vasculitis, Behçet’s syndrome, disseminated fungal infection, epidermolysis bullosa acquisita, and carcinoma [1, 3, 8–10]. Achalasia should also be ruled out with motility testing if symptoms are classic; however, abnormal motility has been described even in patients with inactive CD for unclear reasons and esophageal CD can present like pseudoachalasia [10]. Tuberculosis can be ruled out when biopsies are stained for acid-fast organisms.

CD is known to affect any portion of the gastrointestinal tract; however, the distal third of the esophagus alone is the most common esophageal manifestation (80 %) [3]. Radiographic studies can show ulcers or strictures, but can be normal in up to 50 % of patients with esophageal CD [3]. Other findings include stricture and fistula. The management of these complications entails endoscopic dilation and esophagectomy. For fistula, closure has been described with polymer sealants and infliximab, although many require surgical repair [4]. Given that esophageal CD is a high risk feature indicative of aggressive disease activity, endoscopy should be considered in all patients with CD with upper gastrointestinal symptoms to avoid complications [2, 4].

Medical management is first line when a diagnosis of esophageal CD is made. Currently, the European Crohn’s and Colitis Organisation (ECCO) consensus guidelines suggest esophageal CD should be treated with a proton pump inhibitor, in addition to steroids and thiopurines or methotrexate [11]. Treatment options have historically included 5-aminosalicylic acid preparations, although it is unclear how effective they are because their metabolites are not active in the proximal gastrointestinal tract [8, 9]. Corticosteroids, systemic as well as topical, such as swallowed aerosolized budesonide, have also been described [3, 5, 9]. Immunomodulators including azathioprine and 6-mercaptopurine have also been described; they are steroid-sparing and treat more distal disease if present [5, 9]. There is one case report on the successful use of thalidomide [7]. There are been several case reports commenting on the use of biologics for esophageal CD in recent years as these drugs have become a cornerstone of inflammatory bowel disease treatment. ECCO guidelines recommend a lower threshold for starting TNF-α inhibitors for esophageal involvement than for disease elsewhere owing to the poor prognosis associated with this site [11]. Use of infliximab, adalimumab, and ustekinumab has been associated with rapid improvement on repeat endoscopy [2, 6, 12, 13]. These findings imply that repeat endoscopy should be performed in patients with persistent symptoms to rule out other causes or to confirm persistent disease activity. Optimization of medical therapy is important to minimize complications such as stricture and fistula formation. Patients with persistent dysphagia due to strictures may require dilatation, stent placement, or ultimately surgery for management [3]. Patients are usually also started on a proton pump inhibitor, which can help symptoms but whose role in mucosal healing is unclear.

Tofacitinib, a selective oral inhibitor of JAK 1 through 3, is currently approved for use in patients with rheumatoid arthritis. Inhibition of JAK1 and JAK3 blocks signaling for many cytokines, including IL-2, IL-4, IL-6, IL-7, IL-9, IL-15, IL-21, and IFN-γ, which are involved in immune activation and signaling [14, 15]. Phase 3 trials for the treatment of moderate to severe active ulcerative colitis are currently underway after a positive result from a phase 2 trial that showed significant improvement in primary and secondary endpoints with 15 mg twice daily dosing [14–16]. A separate report from this study demonstrated statistically significant patient reported outcomes when compared to placebo, which correlated with other objective assessments of drug efficacy [15]. A phase 2 trial of tofacitinib for moderate to severe CD published in 2014 was unable to demonstrate an improvement in clinical response or clinical remission when compared to placebo, although they reported it was unclear whether this was a true negative result or related to a high placebo response rate [14, 17]. Decreases in objective markers of inflammation including C-reactive protein and fecal calprotectin suggest that tofacitinib did have biologic activity in this study in patients with CD [14].

One recent retrospective case series, the largest in esophageal CD, reported that categorization of treatment should be based on disease behavior—inflammatory, stricturing, and fistulizing [2]. It is unclear from the literature if disease activity in the esophagus mirrors more distal disease activity or phenotype, although a similarity has been noted on endoscopy [4]. Interestingly, eight patients were on TNF-α inhibitors at the time of diagnosis, and had a clinical response with dose adjustment, adding another agent, or switching TNF-α inhibitors [2]. Our patient had already had unsuccessful trials of other medications for distal disease, thus precluding their use when her esophageal CD was diagnosed. Switching to another biologic has been described and should be of consideration if there is no improvement with intensification of the current therapy.

## Conclusions

Esophageal CD may be a difficult diagnosis to obtain and requires a crossroads between clinical, endoscopic, histological, and radiographic data. This case highlights the use of tofacitinib as an alternative therapy for patients with esophageal CD, and should be a consideration especially in patients who do not benefit from TNF-α inhibitor use. This case also highlights the ability of patients to develop esophageal CD during TNF-α inhibitor therapy, a phenomenon previously described in the literature [5].

## Abbreviations

AFB: Acid-fast bacillus
CD: Crohn’s disease
ECCO: European Crohn’s and Colitis Organisation
IFN: interferon
IL: interleukin
JAK: janus kinase
TNF-α: tumor necrosis factor-α
